# Harnessing extremophilic carboxylesterases for applications in polyester depolymerisation and plastic waste recycling

**DOI:** 10.1042/EBC20220255

**Published:** 2023-08-11

**Authors:** Gwion B. Williams, Hairong Ma, Anna N. Khusnutdinova, Alexander F. Yakunin, Peter N. Golyshin

**Affiliations:** Centre for Environmental Biotechnology, School of Natural Sciences, Bangor University, Deiniol Road, Bangor LL57 2UW, U.K.

**Keywords:** extremophiles, extremophilic, plastics, polyester, polyesterases, thermophiles

## Abstract

The steady growth in industrial production of synthetic plastics and their limited recycling have resulted in severe environmental pollution and contribute to global warming and oil depletion. Currently, there is an urgent need to develop efficient plastic recycling technologies to prevent further environmental pollution and recover chemical feedstocks for polymer re-synthesis and upcycling in a circular economy. Enzymatic depolymerization of synthetic polyesters by microbial carboxylesterases provides an attractive addition to existing mechanical and chemical recycling technologies due to enzyme specificity, low energy consumption, and mild reaction conditions. Carboxylesterases constitute a diverse group of serine-dependent hydrolases catalysing the cleavage and formation of ester bonds. However, the stability and hydrolytic activity of identified natural esterases towards synthetic polyesters are usually insufficient for applications in industrial polyester recycling. This necessitates further efforts on the discovery of robust enzymes, as well as protein engineering of natural enzymes for enhanced activity and stability. In this essay, we discuss the current knowledge of microbial carboxylesterases that degrade polyesters (polyesterases) with focus on polyethylene terephthalate (PET), which is one of the five major synthetic polymers. Then, we briefly review the recent progress in the discovery and protein engineering of microbial polyesterases, as well as developing enzyme cocktails and secreted protein expression for applications in the depolymerisation of polyester blends and mixed plastics. Future research aimed at the discovery of novel polyesterases from extreme environments and protein engineering for improved performance will aid developing efficient polyester recycling technologies for the circular plastics economy.

## Introduction

Global plastics production has increased 20-fold since the 1960s, reaching over 390 million tonnes in 2021 [1]. Plastics production is expected to double over the next 20 years demonstrating a rapidly rising demand for plastic products. Plastic production continues to rise yearly, with 390.7 million metric tonnes (Mt) of plastics produced in 2021, of which 352.3 Mt were from petroleum-based synthetic plastics, and estimates predicting a quadrupling of production to 1,800 Mt of resin per year by 2050 [1,2]. A significant fraction of consumer plastics encompasses polyesters, particularly polyethylene terephthalate (PET), which accounted for an estimated 24.2 Mt (6.2%) of total global production in 2021 [1,2] (Table 1). Polyesters are found in packaging, textiles, automotive parts to name a few [1,3]. Despite the conventional recycling streams commonly processing polyester (PET) waste with up to 60% of consumer waste reaching recycling plants; recycled PET accounts for just 24% of PET products in Europe [4].

**Table 1:** Common polyester plastics and their characteristics

Polymers	Monomer structure	*T*_g_ (°C)	*T*_m_ (°C)	Applications	Ref.
***Major Polyesters***					
Poly(ethylene terephthalate) (PET)	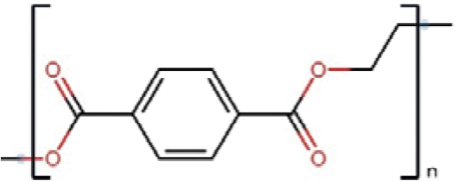	40	250–265	Packaging, textiles and photovoltaics	[55,161,162]
Poly(butylene terephthalate) (PBT)	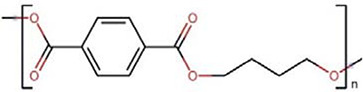	55–65	225	Electrical insulation and automotive manufacture	[163]
Polylactic acid (PLA)	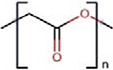	45–60	150–162	Biodegradable packaging and agriculture	[164]
***Other polyesters***					
Polytrimethylene terephthalate (PTT)	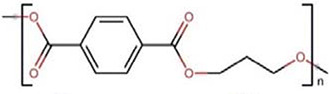	45	228	Fabrics	[165]
Polycaprolactone (PCL)	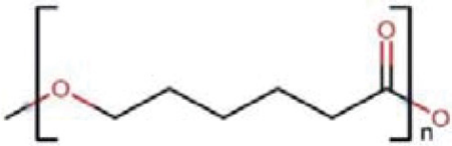	-60	60	Drug delivery	[166]
Polyethylene naphthalate (PEN)	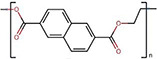	112–120	270	High-performance fibres	[167]
Polybutylene adipate terephthalate (PBAT)	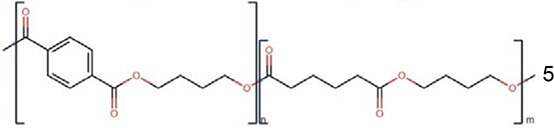	5	170–180	Biodegradable packaging	[168]
Polybutylene succinate (PBS)	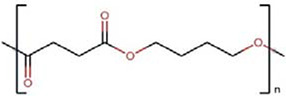	-26	116.4	Disposable tablewear	[169]
Polyglycolic acid (PGA)	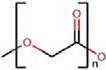	34–40	220–230	Medical suturing	[164]
Polyhydroxyalkanoates (PHA)	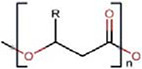	2–8	160–175	Surgical fasteners	[170]
***Related polymers***					
Polyurethane (PUR)	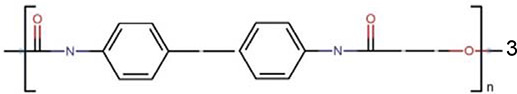	-53	N/A	Upholstery and bedding	[171]

The recalcitrant nature of plastics leads to their prolonged persistence and accumulation across a range of environments [5,6]. Previous studies have shown that between 4.8–12.7 Mt of macroplastics and 1.5 Mt of microplastics are entering oceans every year, and there are estimates that nearly 2/3 of all plastics ever produced are ending in landfills or in the environment [2,7,8]. Therefore, plastics recycling is important for reducing environmental pollution, energy consumption, and CO_2_ emission, as well as for the recovery of polymers and conservation of fossil feedstocks [1].

**Figure 1:**
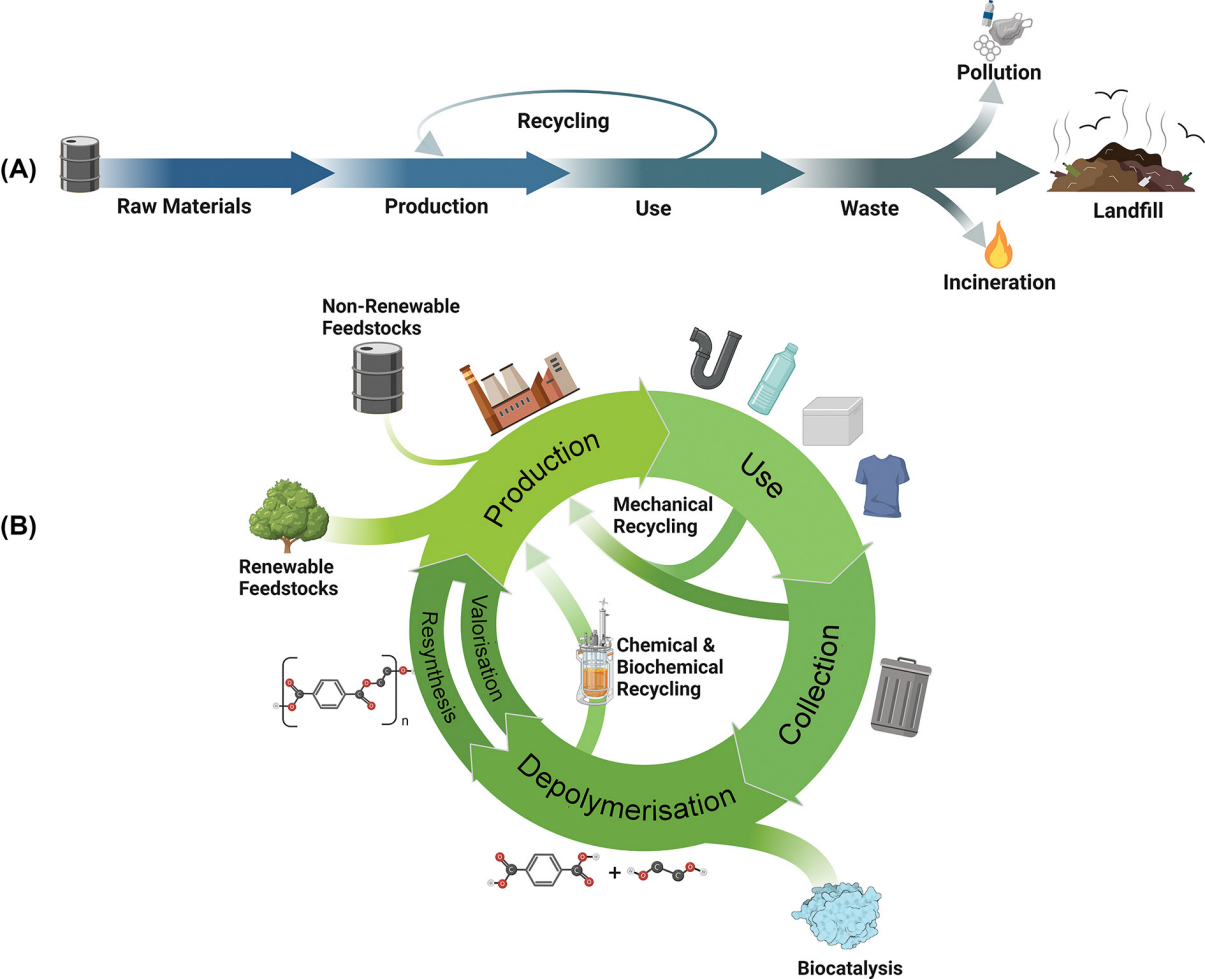
Linear and circular models of plastics economy (**A**) The current linear model of plastic economy where the majority of plastic waste ends up in landfill, litters the environment, or is incinerated. (**B**) The circular plastics economy is based on using renewable feedstocks, improved waste collection, plastic waste recycling to monomers using physical, chemical, and enzyme-based technologies, monomer valorisation or polymer resynthesis, and production of new plastic materials. With biocatalysis we refer to enzyme-based recycling with physical and chemical pre-treatment steps.

Currently, plastics recycling mainly occurs via a mechanical approach based on sorting plastics by polymer type, shredding and melting [9]. However, mixed-polymer plastics and soiled plastics cannot be recycled in this way, leading to a significant fraction of ‘recycled’ plastics being dispensed to landfill [1] or into the environment [10–13] (Figure 1). Moreover, over time we see a ‘downcycling’ of materials recycled in this way, that are suitable for only lower performance applications with every round of recycling [9], thus maintaining the need for *de novo* synthesis of plastics [14]. Therefore, current approaches to plastic waste management (PWM) are evidently unable to deal with the crisis of environmental plastic pollution [15]. To address these issues, a new model of plastics production and reuse is required, encompassing the improved collection of waste, depolymerisation, resynthesis, and valorisation through chemical and biochemical recycling [15] (Figure 1). Creating a closed cycle of plastic materials via the recycling and upcycling of polymers with only minimal input from *de novo* synthesis using petroleum feedstocks will allow the move towards a circular economy of plastics [9] (Figure 1). Furthermore, the valorisation of plastic waste materials is predicted to be a major growth industry for plastics in years to come [16,17]. Polyesters are especially suited for a circular process of production and waste management, due to the presence of ester groups that can be attacked during depolymerisation.

Chemical recycling has been mooted as a more efficient alternative to physical recycling, allowing both resynthesis and upcycling of materials [18]. Primarily, chemical recycling refers to feedstock recycling – whereby waste plastic products are depolymerised becoming feedstock for the next round of synthesis [15,19]. The chemical methods of polyester (PET) recycling, such as methanolysis [20] and glycolysis [21], have been extensively explored [22–26]. However, they are reliant on large thermal inputs [22], elevated pressures [23], and toxic reagents [24–26].

An attractive alternative towards plastic waste recycling is biocatalysis based on using of enzymes as catalysts [27–29]. In recent decades, many sectors such as the pulp and paper [30] or textile industries [31] have replaced traditional catalysts with biocatalysts. However, most biotechnological enzymes, including known plastic-degrading proteins, are derived from mesophilic organisms as can be seen in the Plastic Active Enzyme Database (PAZy) [32]. Therefore, they are active and stable within a narrow range of temperatures making them not applicable for industrial polyester depolymerisation. Thus, an expansion of the enzyme repertoire for more thermostable and robust proteins is required for full-scale utilisation of enzymes in PWM [33,34].

## Extremophilic microorganisms as a source of robust enzymes for polyester recycling

One of the major limitations to widespread adoption of biocatalysis in polyester recycling is that most biotechnological enzymes are of mesophilic origin and exhibit low performance at harsh reaction conditions required for industrial polyester depolymerisation. Although extreme environments present significant challenges to microorganisms, some of them enjoy their life in severities of temperature (-20 to -122°C, pH 0–12.8, salt concentration (>5 M NaCl) and pressure (110 MPa) [35–38]. Such microorganisms (extremophiles) achieve this by evolving a suite of enzymes (extremozymes) enabling them to flourish under conditions. The known biochemical adaptions of extremozymes include an increased hydrogen bonding [39], increased hydrophobicity of protein core [40], reduced charge [41,42], and reduced surface-to-volume ratio [43–46]. Several extremozymes have already been used in molecular biology and biotechnology, whereas other enzymes are currently being developed [47,48]. Thus, extremophilic microorganisms represent an attractive and still a vastly underexplored resource for the mining of biocatalysts for polyester recycling.

Thermophilic microorganisms thrive in hot environments (45–113°C) [38,49,50], and they have evolved various thermostable enzymes. Thermophilic enzymes are especially advantageous for polyester depolymerisation as higher temperatures increase flexibility and accessibility of polyester chains for enzymatic hydrolysis [33]. Archaea are common in thermophilic habitats; however, their enzymes remain largely underexplored compared to bacteria [48]. Thermophilic enzymes retain high activity at elevated temperatures (>60°C) near the melting point (*T*_m_) of polyesters. Furthermore, many enzymes from thermophilic and hyperthermophilic microorganisms show robust performance at 90–103°C [51,52] (near *T*_m_ of some polyesters), and in some cases they retain significant activity at these temperatures for several hours [51,52]. It is hypothesised that the biodegradation efficiency of PET is limited by the accessibility of ester bonds, and that the susceptibility of polymeric chains increases with temperatures [53,54]. Thus, enzymes exhibiting significant activity above the surface glass transition temperature (*T*_g_) of PET (∼40°C) are of high value for applications in polyester recycling [55,56].

Acid-resistant enzymes are also important for polyester depolymerisation, as acid pre-treatment increases the accessibility of polyester chains, and polyester hydrolysis releases organic acids (terephthalic acid for PET) [57,58]. Similarly, alkali-tolerant enzymes are useful for polyester depolymerisation under alkaline conditions or in combination with alkaline PET pre-treatment, which can enhance degradation yields by reducing polymer crystallinity, leading to improved enzyme access to polymer chains [59,60]. Many halophilic enzymes also exhibit significant thermostability and alkali tolerance, whereas psychrophilic enzymes retain high activity at low temperatures (5–15°C) [61–63].

## Discovery of extremophilic enzymes for polyester recycling

Currently, the discovery of novel enzymes is primarily based on three approaches: *in silico* (homology-based) sequence mining, activity-based protein profiling (ABPP), and activity-based screening of metagenomic libraries [64,65].

Homology-based mining of genome and metagenome sequences is generally regarded as the simplest and cheapest approach to enzyme discovery [66–73]. The sequence homology-based approach involves mining publicly available sequence datasets for enzymes of relevance. Recently, this approach was used with great success with the identification of 37 thermostable enzymes with PET degrading activity from public databases [69]. Subsequent analysis using sequence data exploration platforms such as those offered by the Joint Genome Institute [74] and functional prediction software such as HMMER [75] are utilised to mine for known motifs and predict putative protein function based on sequence homology. However, sequence homology-based approaches are limited to identifying known motifs, and therefore they cannot identify novel activity types [76].

ABPP is based on small-molecule probes, which bind specifically to enzyme active sites and ‘tag’ them with different reporter molecules [77–83]. The strength of ABPP as an enzyme discovery tool lies in direct identification of novel enzymes, which have no sequence similarity to known biocatalysts [84,85]. In the field of drug discovery, the application of ABPP was highly successful in recent years [86]. However, despite its potential to provide direct analysis of enzymatic activity [85,92], ABPP remains an underutilised tool in the exploration of extremophilic proteomes for plastics degrading enzymes [65,85].

Enzyme activity (*naïve*) screening of metagenomic gene libraries is a general approach to enzyme discovery based on screening *Escherichia coli* clones expressing metagenomic DNA fragments against different substrates [87,88]. An advantage of such functional screens is their ability to identify new enzymes without relying on sequence homology to already characterised proteins, and thus they can uncover proteins representing fundamentally novel enzyme families [89]. This approach was used by many groups with great success leading to a trove of enzyme discoveries [51,87,90–93]. There are certain limitations to this approach, including a narrow range of hosts, suboptimal protein expression, and reliance on general substrates [76,94]; moreover, the only recent development of metagenome screens directly assaying for plastic biodegradation activity [95] means that more time will be required for such screens to uncover novel classes of polymer-degrading enzymes.

Recently, activity-based metagenome screening approaches have been expanded and complemented by application of microfluidics and flow cytometry [96–99], as well as *in vivo* reporter systems making use of fluorescence biosensors, which allow for semi-quantitative monitoring of PET degradation product formation [99–101]. Overall, a combination of all three outlined methodologies seems to be the most successful approach in search for novel polyesterases.

## Polyester degrading microbial carboxylesterases (polyesterases)

**Figure 2:**
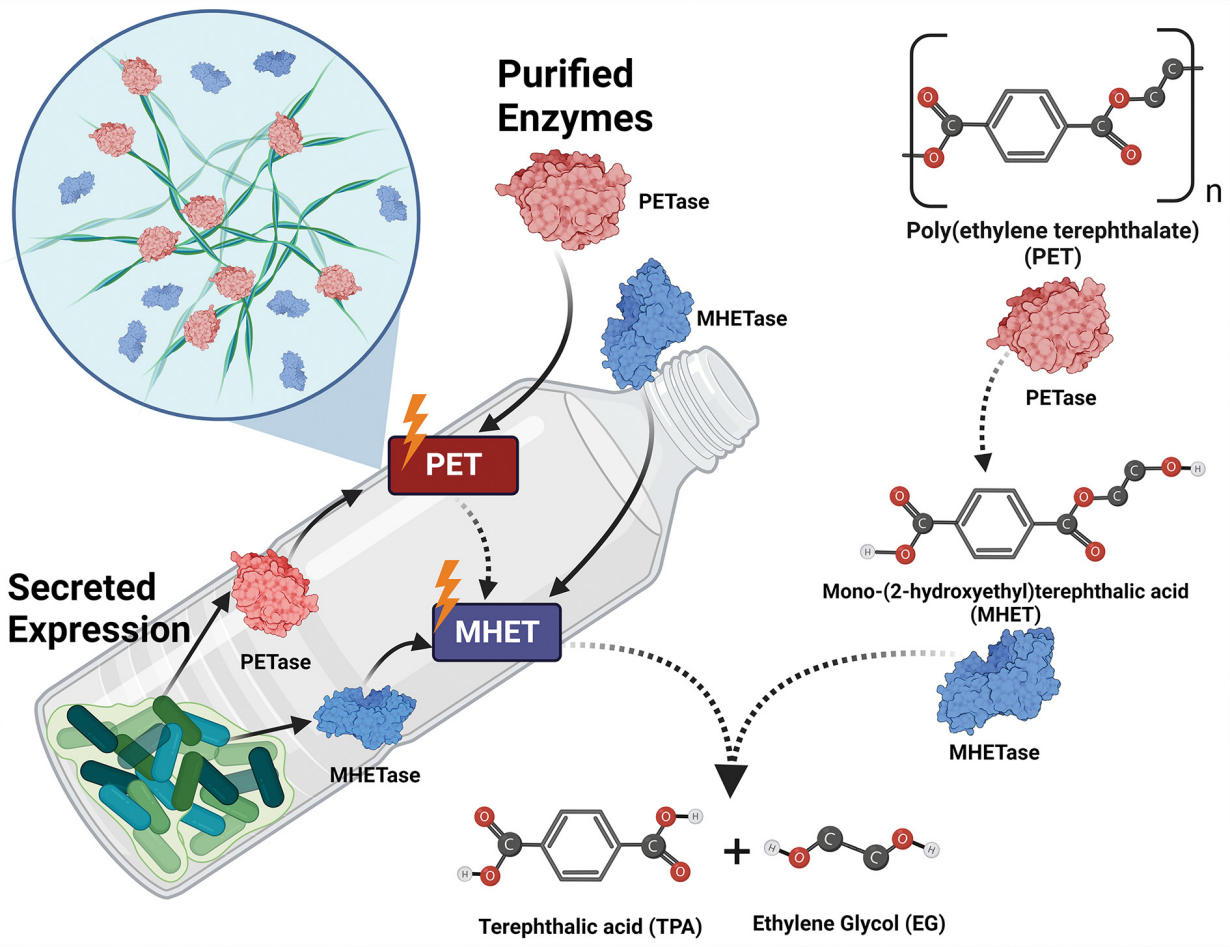
Example applications of enzyme cocktails for polyester degradation The primary approaches of secreted expression (including surface display and direct secretion), and use of purified enzymes in synergistic cocktails to tackle polyesters and their intermediary degradation products are vital for true polyester degradation. Shown are structures of PET with its primary degradation products, *Is*PETase (5XJH) and *Is*MHETase (6QZ4).

Carboxylic-ester hydrolases – carboxylesterases (EC 3.1.1.1), cutinases (EC 3.1.1.74) and lipases (EC 3.1.1.3) – are key targets of enzyme discovery for polyester recycling. To this end, several thermophilic PET hydrolases (PETases) were discovered in the early 2010s including cutinases, LCC (from leaf-branch compost) [102], Tfcut_2 (from *Thermobifida fusca*) [103], HiC from *Humicola insolens* [104,105], and Est119 from *Thermobifida alba* [106] (Table 2). These enzymes exhibited significant thermotolerance with optimal reaction temperatures above the surface *T_g_* of PET (Table 2), with LCC outperforming other enzymes (*T*_opt_ of 65°C) [102,107,108]. In 2016, the mesophilic bacterium *Ideonella sakaiensis* was isolated from a plastics recycling facility, representing the first described microorganism with a 2-enzyme system for PET degradation comprising two carboxylesterases – *Is*PETase and *Is*MHETase [109,110] (Table 2). The hydrolytic activity of *Is*PETase against PET is likely a result of its natural substrate promiscuity rather than of *in situ* evolution, as discussed elsewhere [111]. This enzyme degrades PET to a monoester intermediate, mono(2-hydroxyethyl)terephthalate (MHET), which is hydrolysed by *Is*MHETase to terephthalic acid and ethylene glycol (Figure 2). In addition, thermotolerant PET-hydrolysing activity was also demonstrated in fungal lipases from *Candida antarctica* (CalB) [105,112,113] and *Thermomyces lanuginosus* [114–116].

**Table 2:** Selected biochemically and structurally characterised prominent microbial polyesterases

Enzyme	Source	Uniprot ID	Degraded polyesters	*T*_opt_ (°C)	Structure	PDB ID	Ref.
**LCCut** *(cutinase)*	Leaf-branch compost metagenome uncultured bacterium	G9BY57	PET	65	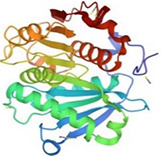	4EB0	[107]
**TfCut_2** *(cutinase)*	*Thermobifida fusca*	Q6A0I4	PET	60	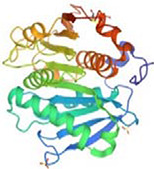	4CG1	[103]
**Est119** *(cutinase)*	*Thermobifida alba*	F7IX06	PET, PBSA, PLA	50	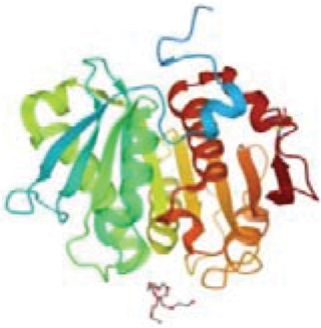	3VIS	[106]
**IsPETase** *(carboxylesterase)*	*Ideonella sakaiensis*	A0A0K8P6T7	PET	40	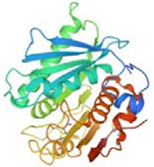	5XJH	[110]
**HiC** *(cutinase)*	*Humicola insolens*	A0A075B5G4	PET, PU-PE	70–80	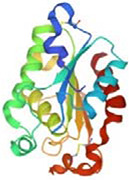	4OYY	[104]

## Engineering polyesterases for enhanced activity and stability

While some wild-type polyesterases (e.g. cutinases LCC [102] and Tfcut_2 [103] (Table 2)) have been shown to exhibit significant PETase activity, there is a great demand for expanding our ‘enzyme toolbox’ by adding novel highly active and robust polyesterases [32]. However, the natural evolution of PETases and other polyesterases is delayed by the recalcitrant nature of polymers making them ‘invisible’ to microorganisms that prefer to use other, easy-to-degrade carbon sources available *in situ*. Nevertheless, natural polyesterases appear to have evolved before the era of the industrial production of synthetic polyesters as indicated by the presence of polyesterase activity in microbial cutinases and in many promiscuous carboxylesterases [117]. Therefore, recent years have seen an explosion in protein engineering techniques applied to PETases including rational design, fusion proteins, directed evolution, surface display, and ‘Plurizymes’ (engineered enzymes with several active sites) [13,59,108,118–132].

The rational design engineering for improving enzyme thermostability and activity is based on detailed knowledge of enzyme structure [133,134]. This engineering strategy can be facilitated by using additional *in silico* approaches, such as molecular docking, analysis of enzyme surface, and structural modelling (AlphaFold2) [108,135]. Amino acid substitutions in the substrate-binding cavity, insertion of new catalytic residues, replacing the metal binding sites with disulfide bonds has been shown to have various effects on enzyme activity and stability [88,110,136]. Rational design has already been applied to improve the thermostability of the relatively thermotolerant LCC cutinase resulting in several enhanced variants with the LCC^ICCG^ protein degrading 90% of amorphous or pre-treated PET within 10 h at 72°C [56,108]. Next, the recovered monomers were used to produce virgin PET and new bottles, thus closing the recycling loop. Another engineering strategy for improving the thermostability of the *T. fusca* cutinase *Tf*Cut2 and homologous PETases involved substituting the Ca^2+^-binding site near the enzyme active site with a salt bridge or disulfide bond [108,131,137,138].

Recent advances in structural bioinformatics have led to the development of computational tools for enzyme engineering for improved stability, activity, and substrate specificity [139]. The GRAPE approach (greedy accumulated strategy for protein engineering) involved a systematic clustering analysis and selection of beneficial mutations from a computationally derived protein library of *Is*PETase and produced the DuraPETase variant with enhanced thermostability and PET degradation [125]. Last year, a structure-based, machine learning approach was applied to improve the PET-hydrolysing activity of *Is*PETase producing FAST-PETase with superior activity [120,121]. Recent protein design studies with *Is*PETase also reported the development of more stable and active variants using rational protein engineering (ThermoPETase) or directed evolution (HotPETase) [119,140]. Finally, ancestral sequence reconstruction was used to trace the evolutionary origin of *Is*PETase from ancient cutinases and generated several variants with improved activity and stability [141].

Another promising strategy for improving enzymatic PET depolymerisation is based on covalent fusion of PETases to various substrate-binding domains including the cellulose-binding domains (from *Cellulomonas fimi* and *Trichoderma reesei*), the polyhydroxyalcanoate-binding module from the *Alcaligenes faecalis* PHA-depolymerase, the chitin-binding module from the *Chitinoliticbacter meiyuanensis* chitinase CmChi1, and fungal hydrophobins [137,142–144]. Similar to cellulases, the polymer binding modules are suspected to stimulate PETase binding to PET at low to intermediate substrate loading levels.

## Additional approaches for improving enzymatic PET depolymerisation

Microorganisms are known to secrete synergistic enzyme mixtures to degrade recalcitrant natural polymers, such as cellulose, hemicellulose, and chitin [145,146]. Natural microbial communities degrade various polymers using even more complex enzyme mixtures, which show higher efficiency compared with single enzymes [55,105,147–151]. These enzyme cocktails usually include two types of enzymes, the first acting on polymeric substrates and producing various oligomeric products and the second degrading oligomeric intermediates to monomers. The discovery of a two-enzyme PET degrading system from *I. sakaiensis* comprising *Is*PETase and *Is*MHETase suggests that these multienzyme systems also have capacity to act promiscuously and synergistically to degrade synthetic polyesters [109,148]. This also implies that synergistic multienzyme cocktails can be designed for the depolymerisation of synthetic polyesters and complex polymer blends. In this respect, the combinations of wild type or thermostable variants of *Is*PETase and *Is*MHETase demonstrated synergistic activity in the conversion of amorphous PET films to terephthalic acid and ethylene glycol, whereas the *Is*PETase–*Is*MHETase fusion showed even better performance [55,147,148]. Similarly, combinations of the promiscuous *T. fusca* carboxylesterase *Tf*Ca (exhibiting both BHETase and MHETase activities) with various polyester hydrolases were amongst the first dual enzyme systems for PET hydrolysis reported, and showed significantly improved activity compared with single enzymes: the use of immobilised *Tf*Ca in concert with *Tf*Cut2 and LCC exhibited a 91 and 104% increase in degradation products, respectively [149], and recent work combining an engineered variant of *Tf*Ca with *Is*PETase penta-mutant [138] to create a dual enzyme system resulted in an up to 14-fold increase in TPA production compared with the PETase alone [152]. Likewise, the combination of the *Humicola insolens* cutinase HiC and *Candida antarctica* lipase CalB catalysed complete PET hydrolysis with HiC acting as a PETase and CalB as a MHETase [104,105,112]. PET degradation performance of *Is*PETase was also improved by the addition of free hydrophobins, catalytically inactive lytic polysaccharide monooxygenase PcAA14A from *Pycnoporus coccineus*, and a zwitterionic Lys-Glu polymer [122,143,153].

**Figure 3:**
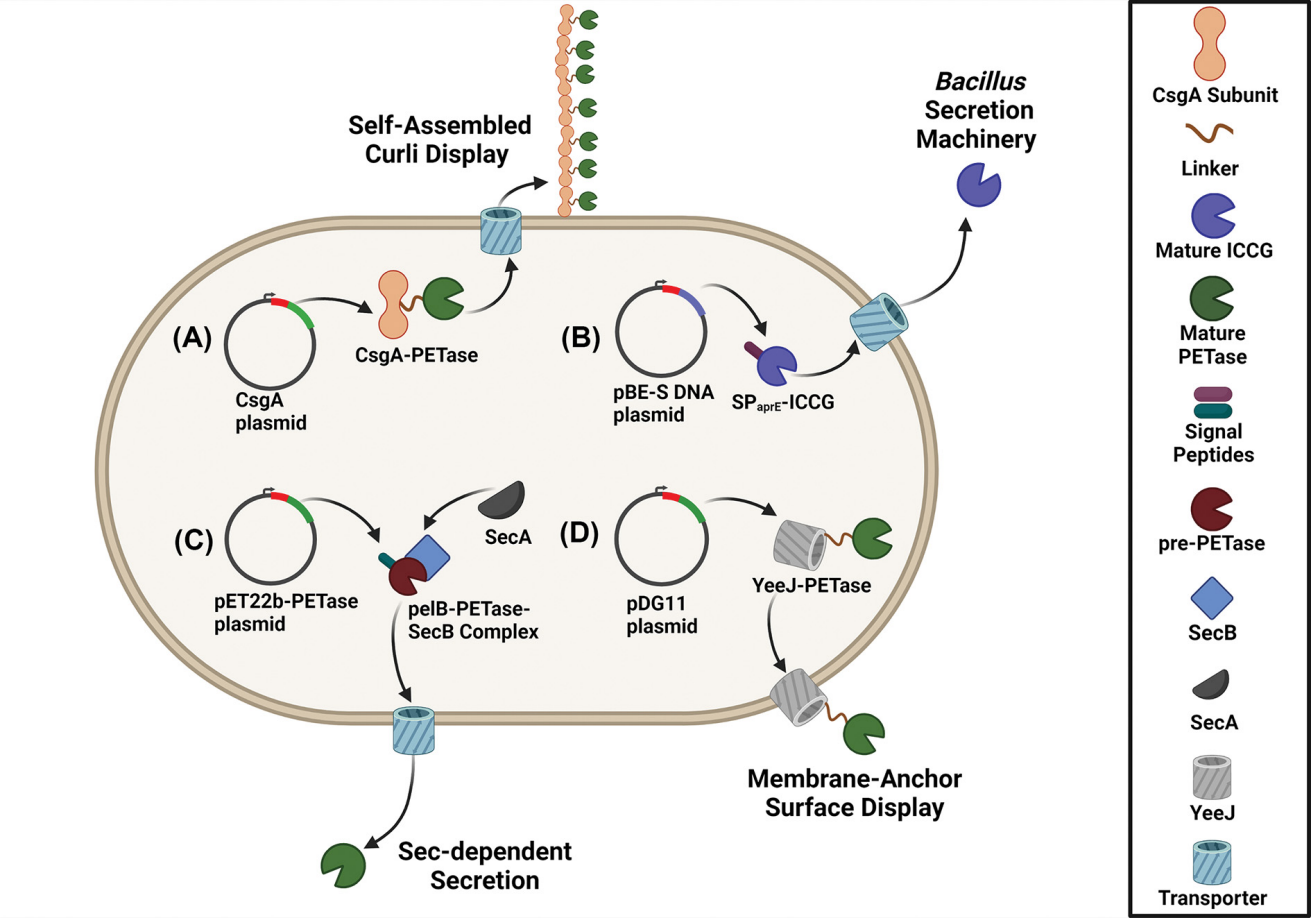
Approaches for whole cell biocatalysis for enzyme-based plastics recycling (**A**) Curli display; (**B**) B*acillus* spp. machinery-based secretion; (**C**) Sec-dependent secretion; (**D**) Membrane anchoring.

Enzyme immobilization represents a powerful tool for increasing enzyme stability and its life span, as well as for reducing enzyme costs via the biocatalyst reuse. In this regard, immobilization of *Is*PETase on Co_3_(PO_4_)_2_ nanoparticles has been shown to increase the enzyme lifetime by 75% [154]. Furthermore, the silica-immobilised PETase was successfully applied for wastewater treatment [154,155], whereas magnetic nanoparticles-tagged PETase was used for removal of PET microplastic [156]. Protein surface display represents another strategy for enzyme immobilisation, which is based on a functional display of target enzymes through fusion to various secreted proteins [59,123,124,127,129] (Figure 3). Moreover, surface display allows for a streamlining of conventional functional screening assays [157]. The *E. coli* protein CsgA represents the building block of curli nanofibers assembled on the cell surface enabling functional expression and immobilisation of target proteins fused to CsgA [128,150]. The CsgA-*Is*PETase fusion protein (‘BIND-PETase’) was secreted by *E. coli* cells forming self-assembling fibres (Figure 3) [123] and degraded 9.1% of postconsumer PET in seven days [123]. The co-display of *Is*PETase with the hydrophobin HFB1 from *Trichoderma reesei* demonstrated enhanced degradation of both high- and low- crystallinity PET substrates [59]. In both cases, the biocatalysts also displayed excellent durability, with BIND-PETase remaining active for 7 days at 30°C and stable at 4°C for at least 30 days. The co-display system retained full activity after seven days at 30°C, whereas free *Is*PETase lost 40% of its activity after one day [59].

Likewise, secreted expression of soluble polyesterases can reduce the enzyme costs for enzymatic polyester recycling. Enzyme secretion methods are based on covalent fusion of target enzymes to host-specific signal peptides or secreted proteins [13,126,130,158,159]. Several groups have reported on using the *E. coli* Sec-dependent pathway with the *Is*PETase–PelB fusion showing high secretion and degradation of PET at 30°C [160]. The protein secretion machinery of *Bacillus subtilis* was used to produce extracellular LCC^ICCG^ fused with the signal peptide SP_aprE_, which showed high PET degradation (approximately 7%) after 8 days at 70°C [130].

## Concluding remarks

For the effective degradation of highly crystalline post-consumer plastic waste several important elements are required. Firstly, thermal and acid pre-treatment of plastic waste materials to make them more accessible for degradation. Secondly, the single enzyme model must be re-considered towards implementation of enzyme cocktails for catalytic breakdown of polymers, intermediary products, and additives present in plastic materials.

In both cases, the currently sparse enzymatic toolkit requires upgrading to include stable and robust enzymes with a high degree of substrate promiscuity and active in the broad range of physico-chemical conditions. In that context, extremophilic microorganisms represent a critically under-explored resource to enzyme bioprospecting. Furthermore, the naturally evolved wild-type enzymes can be further improved using protein engineering. Engineered natural and artificial enzymes represent a true shift in the bioprocessing of plastic waste and allow for cost effective methods of material recycling, thereby enabling the move towards the circular economy.

## Summary

- A significant progress has been achieved in the past two decades in discovery and characterisation of polyester-active enzymes, in particular, using activity-centred metagenomics.
- A number of ground-breaking studies on engineering of polyester-active enzymes have delivered enzyme variants active against recalcitrant polyesters.
- Important studies on the development of application of whole-cell catalysts, enzymatic cocktails, enzyme fusion with substrate-binding domains, and surface display have been conducted.
- Despite the importance of high-temperature-active, thermostable and solvent-resistant biocatalysts, extremophilic, and particularly, thermophilic microorganisms have largely been overlooked as a potential source of such enzymes.

## Open Access

Open access for this article was enabled by the participation of Bangor University in an all-inclusive *Read & Publish* agreement with Portland Press and the Biochemical Society under a transformative agreement with JISC.

## Abbreviations

ABPP: activity-based protein profiling
LCC: leaf-branch compost cutinase
MHET: mono-(2-hydroxyethyl)terephthalic acid
Mt: million metric tonnes
PET: polyethylene terephthalate
PETase: PET hydrolase
PWM: plastic waste management
